# Resilience of Key Biological Parameters of the Senegalese Flat Sardinella to Overfishing and Climate Change

**DOI:** 10.1371/journal.pone.0156143

**Published:** 2016-06-09

**Authors:** Kamarel Ba, Modou Thiaw, Najih Lazar, Alassane Sarr, Timothée Brochier, Ismaïla Ndiaye, Alioune Faye, Oumar Sadio, Jacques Panfili, Omar Thiom Thiaw, Patrice Brehmer

**Affiliations:** 1 Institut Sénégalais de Recherches Agricoles (ISRA)/Centre de Recherches Océanographiques de Dakar-Thiaroye (CRODT), Dakar, Sénégal; 2 Université Cheikh-Anta-Diop (UCAD)/Institut Universitaire de Pêche et d’Aquaculture (IUPA), Dakar, Sénégal; 3 Department of Fisheries, University of Rhode Island East Farm, Kingston, Rhode Island, United States of America; 4 Institut de Recherche pour le Développement (IRD), UMR 195 Lemar, Dakar, Sénégal; 5 Institut de Recherche pour le Développement (IRD), UMR Marbec, Université Montpellier 2, Montpellier Cedex 5, France; Hellenic Centre for Marine Research, GREECE

## Abstract

The stock of the Senegalese flat sardinella, *Sardinella maderensis*, is highly exploited in Senegal, West Africa. Its growth and reproduction parameters are key biological indicators for improving fisheries management. This study reviewed these parameters using landing data from small-scale fisheries in Senegal and literature information dated back more than 25 years. Age was estimated using length-frequency data to calculate growth parameters and assess the growth performance index. With global climate change there has been an increase in the average sea surface temperature along the Senegalese coast but the length-weight parameters, sex ratio, size at first sexual maturity, period of reproduction and condition factor of *S*. *maderensis* have not changed significantly. The above parameters of *S*. *maderensis* have hardly changed, despite high exploitation and fluctuations in environmental conditions that affect the early development phases of small pelagic fish in West Africa. This lack of plasticity of the species regarding of the biological parameters studied should be considered when planning relevant fishery management plans.

## 1. Introduction

In Senegal (West Africa), coastal pelagic fishes are the most important marine resource, accounting for about 64% of annual catches [1]. Small pelagic fishes, such as sardinella (*Sardinella aurita* and *Sardinella maderensis*), have long been known to play a key role in the food supply for local communities. The catches of marine fisheries reached 448,000 t in 2012, the highest on record in Senegal [2]. More than 288,000 t of the catch was made up of small pelagic fish, of which 86% were sardinella (119,000 t for *S*. *aurita* and 128,700 t for *S*. *maderensis*) [2]. Sardinella are short-lived, zooplankton feeders with similar shape and size (maximum fork-length ~30 cm). Sardinella stocks have been considered to be overexploited since 2006 [3] by small-scale fishing canoes, a few local purse seiners, large foreign fishing vessels operating within the framework of bilateral agreements and often illegal, unreported, unregulated (IUU) fishing fleets [4, 5].

*S*. *aurita* undertakes extensive north/south seasonal migrations, whereas *S*. *maderensis* is less migratory [6, 7, 8, 9], suggesting that this species is able to adapt more readily to environmental variations. The trophic and ontogenetic migrations of sardinella are related to the strong seasonal variations in the southeastern edge of the Canary Upwelling System, an eastern boundary upwelling current [7, 8]. Sardinella are found along the north and west African coast from the Mediterranean Sea to Cape Frio in Angola, mainly in coastal upwelling areas off northwest Africa (10–26°N), in the Gulf of Guinea off Ghana and the Ivory Coast and in the south from Gabon to Angola (0–18°S) [6]. This paper focuses on the northwest population.

Past studies described the *S*. *maderensis* condition index, reproduction and growth based on data collected more than 25 years ago [6–9]. This study updated these parameters using more recent data from small-scale fisheries and published datasets to detect any changes that may have occurred as a result of climate change and/or over-fishing. The amount of food available from the environment may well change the amount of food reserves fish can build up or the energy they can invest in growth or reproduction [10]. Adaptive responses to overfishing have already been shown for various species, for example by reproducing earlier (e.g. smaller length at first maturity) [11].

The results of this study were compared and discussed in relation to relevant past studies in various north tropical Atlantic areas.

## 2. Materials and Methods

### 2.1. Study area

**Fig 1:**
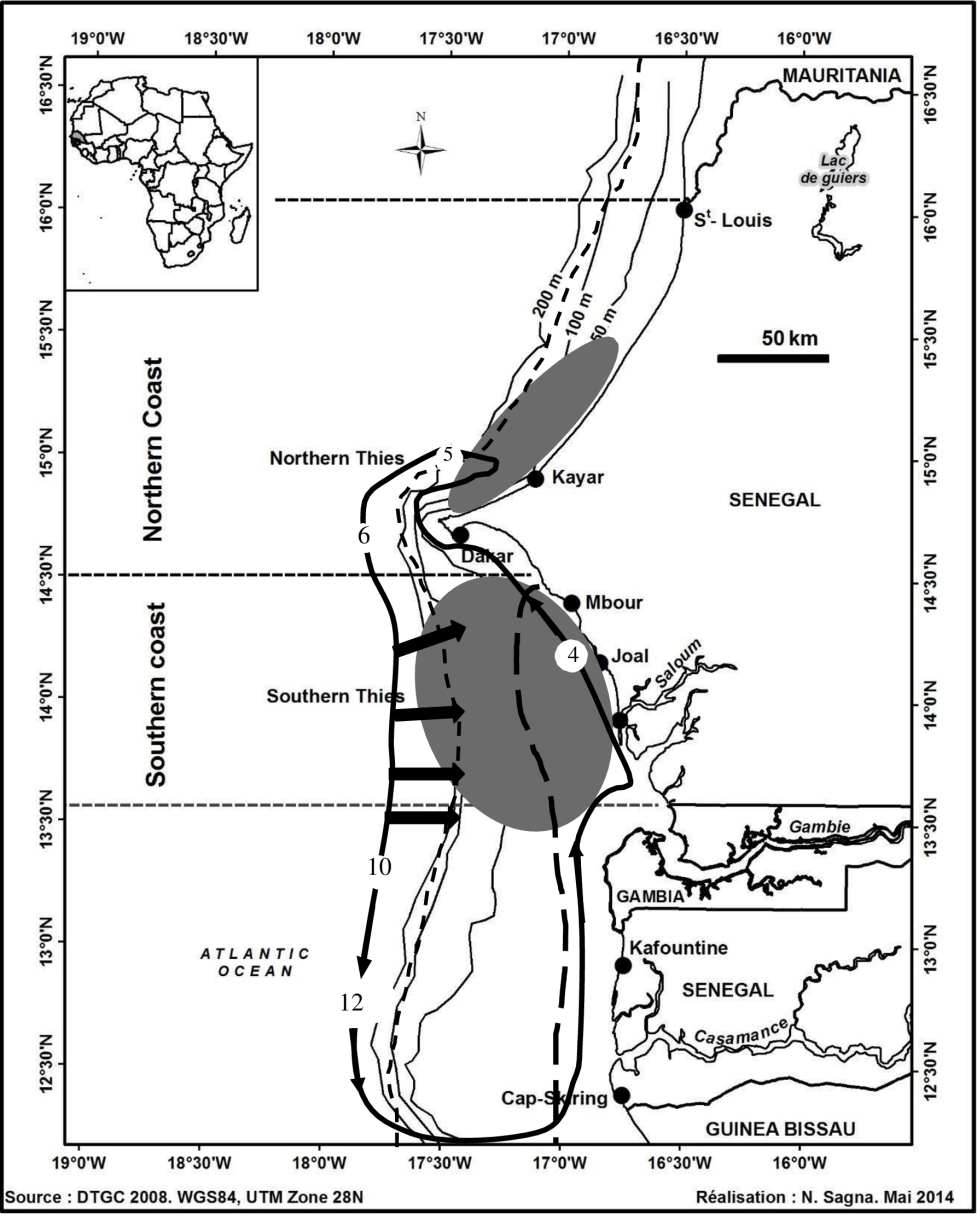
Map of the study area, fishing areas (dark ellipses) covered by sampling sites (Kayar, Mbour, and Joal) and overlay of spatial distribution and migration pattern of *Sardinella maderensis* (adapted from [13]). “Grande Côte” is the northern part (Dakar to Saint-Louis including Kayar) with a narrow continental shelf and “Petite Côte” is the southern part (Dakar to Sine-Saloum) with a wider continental shelf. Casamance is further south between Gambia and Guinea-Bissau. The nursery areas are delimited by a long dashed line (starting at the coast) and the spawning areas are delimited by a continuous bold line with arrows and numbers (months).

The Senegalese Exclusive Economic Zone (EEZ) (West Africa) extends from 18° 00’ N and 20° 00’ W to 12° 15’ N and 16° 30’ W. The Cape Verde Peninsula divides the EEZ into two areas that have distinct topographic characteristics (Fig 1). In the northern part of the zone, the continental shelf is fairly narrow, with the edge running north–north-east. In the southern part of the zone, the continental shelf widens and the edge runs north to south. Senegal has a cold season (November to April) and a hot season (May to October) [9]. The monthly AVHRR Pathfinder sea surface temperature (SST) records (http://coastwatch.pfeg.noaa.gov) with a resolution of 5 km show that the mean SST along the northern and southern coast increased by 0.03°C per year over the 1985–2009 period [12]. Furthermore, the mean SST was higher along the southern coast (24.55°C ± 3.06°C) than the northern coast (23.41°C ± 2.85°C).

Seasonal upwelling occurs along the Senegalese coast between November and May, maintaining a relatively low sea surface temperature depending on the strength of the wind (~15–18°C) [13]. The upwelling becomes permanent further to the north (in Mauritania). This upwelling is closely related to atmospheric dynamics and is determined by the seasonal changes in the direction of the trade winds associated with the migration of the Intertropical Convergence Zone (ITCZ) [14]. The trade winds stop in Senegal from June to October and a moderate northward current brings warm (> 25°C) water to the coast, which can be seen as an extension of the North Equatorial Countercurrent.

### 2.2. Biological data

Flat sardinella catches landed by small-scale fisheries were sampled monthly from March 2012 to February 2013. Purse seine and gill nets were the main fishing gear used. This study sampled only specimens caught using purse seine nets to have the same fishing gear selectivity for monthly comparisons of the length distribution. When available, up to 500 individuals were sampled from the three main landing sites: Kayar, Mbour and Joal (Fig 1). Interviews with fisherman (36 interviews; with one per month per site) performed during the sampling campaign, indicated that canoes fishing with purse seine nets at Kayar covered the area 15° 50’ N to 14° 80’ N. Mbour and Joal had overlapping fishing zones ranging from 14° 30’ N (in Senegal) to 13°48’ N (in Gambian waters).

The fork length (FL) of the fish sampled was measured to the nearest 1 mm and the body weight (W) was determined to the nearest 0.01 g. One hundred fish were dissected and sexed each month at each site. The maturity stages were determined macroscopically using the Fontana [15] maturity scale (Table 1). Eviscerated fish and gonads were also weighed.

**Table 1:** A brief description of the maturity stages according to [15], used in this study.

Stage	State	Description
**I**	Virgin	Sexual organs very small, situated close to vertebral column. Ovary and testis about 1/3rd length of body cavity. Testis and ovary transparent, colorless or grey. Eggs not visible to naked eye.
**II**	Maturing virgin	Testis and ovary translucent, grey- red. Length of gonads 1/2, or slightly more, of length of ventral cavity. Individual eggs can be seen with magnifying glass.
**III**	Ripening	Testis and ovary opaque, reddish with blood capillaries. Occupy about 1/2 of ventral cavity. Eggs visible to naked eye as whitish granular material.
**IV**	Ripe	Testis reddish-white, no milt produced under pressure. Ovary orange-red in color with conspicuous superficial blood vessels. Eggs clearly discernible, opaque. Testis and ovary occupy about 2/3rds of ventral cavity. Large transparent, ripe ova visible.
**V**	Spawning	Sexual organs fill ventral cavity. Testis whitish- creamy, soft. Drops of milt produced under slight pressure. Eggs completely round, some already translucent and ripe.
**VI**	Spent	Ovary and testis shrunken to about 1/2 length of body cavity. Ovary may contain remnants of disintegrating opaque and ripe ova, darkened or translucent. Testis bloodshot and flabby.
**VII**	Resting	No opaque eggs left in ovary. Testis and ovary red and empty. A few eggs in state of resorption.

### 2.3. Allometric relationships and condition factor

Length-weight relationships of the form:

$$W=a\times {FL}^{b}$$ (Eq 1)

were used [16], where W is the weight and FL is the fork length as defined in section 2.2, and a and b are estimated parameters.

The condition factor was defined using the Le Cren index [16] from:

$$CF=\frac{W_{obs}}{W_{th}}$$ (Eq 2)

where

W_obs_ is the observed weight and W_th_ is the theoretical weight (the weight predicted by Eq 1).

### 2.4. Estimating the von Bertalanffy growth parameters

The available length frequency data were grouped into 2 cm class intervals arranged by month from March 2012 to February 2013 (Table 2). The data were then analyzed using the R ELEFAN package, a recently updated Electronic LEngth Frequency ANalysis method (Daniel Pauly, unpublished). The von Bertalanffy Growth Function (VBGF) modified to allow for seasonal growth oscillations [17] was used to describe the growth of *S*. *maderensis*:

$$L_{t}=L_{\infty }\times (1-e^{-K\times (t-t_{0})+{St}_{s}-{St}_{0}})$$ (Eq 3)

where

L_t_ is the predicted length at age t, L_∞_ is the asymptotic length, the mean length that fish in a given population would reach if they were to grow continuously, K is the curvature parameter of the VBGF [18] and t_0_ is the “age” of the fish at zero length if they had always grown as predicted by the Equation, and where:

$${St}_{s}=\frac{C\times K}{2\pi }\sin\left(2\pi (t-t_{s})\right)$$ (Eq 3a)

$${St}_{0}=\frac{C\times K}{2\pi }\sin\left(2\pi (t_{0}-t_{s})\right)$$ (Eq 3b)

$$\text{and}\;t_{s}=WP-0.5$$ (Eq 3c)

where

t_s_ is the start of every seasonal cycle with respect to t = 0, C is the amplitude of seasonal growth oscillations [18] and WP is the winter point (the period of the year when growth is most reduced). C was fixed at 0.1 and WP at 0.15, in this study.

**Table 2:** Size distribution of *Sardinella maderensis* specimens (n = 7708) collected in Senegalese waters from March 2012 to February 2013.

Midpoint Length (cm)	15/03/2012	15/04/2012	15/05/2012	15/06/2012	15/07/2012	15/08/2012	15/09/2012	15/10/2012	15/11/2012	15/12/2012	15/01/2013	15/02/2013
**13**	0	0	0	0	0	3	0	11	0	0	0	0
**15**	0	0	0	0	1	0	0	6	0	5	0	0
**17**	0	0	0	0	6	26	1	16	2	56	49	3
**19**	0	0	0	0	7	53	54	3	130	71	48	5
**21**	0	0	2	4	14	53	25	21	259	73	30	15
**23**	53	23	48	14	271	220	263	160	169	258	165	238
**25**	154	218	213	148	387	145	317	259	160	235	383	254
**27**	140	235	131	222	96	41	106	66	100	50	121	31
**29**	120	26	103	128	18	13	13	10	5	2	10	4
**31**	8	4	56	38	0	1	2	0	1	0	0	0
**Total**	475	506	553	554	800	555	781	552	826	750	806	550

ELEFAN determines the growth curve passing through a maximum number of peaks by adjusting L_∞_ and K to maximize the goodness of fit R_n_:

Rn=10ESPASP10 (Eq 4)

where ASP (available sum of peaks) is the sum of all values of available peaks, and ESP (explained sum of peaks) is the sum of all peaks and troughs through which the growth curve passes [19–20]. The growth performance index *φ*’ [21] was used to evaluate growth parameter estimates, as its values are similar for the same species [22]. *φ*’ is defined as:

$$\varphi^{\prime }=\log_{10}\;K+2\times \log_{10}\;L_{\infty }$$ (Eq 5)

Because the value of t_0_ cannot be estimated from length-frequency data, a very approximate value of t_0_ was estimated by substituting L_∞_ (in cm) and K (year^-1^) in the following Equation [23]:

$$\log_{10}(-t_{0})\approx -0.3922-0.2752\times \log_{10}\;L_{\infty }-1.038\times \log_{10}\;K$$ (Eq 6)

### 2.5. Reproductive biology: sex ratio, sexual maturity, and spawning periods

Size-dependent sex ratios were computed from monthly sample data and a chi-square test was used to assess seasonal variations in the sex ratio of *S*. *maderensis*.

The mean length at first maturity (L_50_) was estimated using only samples collected during the reproductive periods. L_50_ is the length at which 50% of the individuals are mature (≥ stage III, maturing, on the maturity scale), as estimated using the logistic function:

$$\%M=\frac{100}{1+e^{-a(FL-L_{50})}}$$ (Eq 7)

where “%M” is the percentage of mature fish,”FL” is the mid-point of each length class and”a” and”L_50_” are estimated parameters. The model was fitted using R software and least squares (quasi-Newton method).

The gonadosomatic index (GSI) is useful for analyzing the state of sexual maturation of female and male fish gonads. GSI was calculated for each sampled individual based on [24], where:

$$GSI=\frac{w_{g}}{W_{Ev}}\times 100$$ (Eq 8)

where w_g_ is gonad weight and W_Ev_ is eviscerated fish weight.

### 2.6. Statistical analysis

All statistical analyses were performed using the “stats”, “pgirmess” and “agricolae” R packages, with a significance level of α < 0.05. In general, the statistical analyses were taken from [25–27].

The Shapiro test was used the assumption of normality and Bartlett’s test was used for the assumption of homogeneity of variance as Student’s t-test assumes that the observations within each group are normally distributed and that the variances are equal in both groups. Student’s t-test was used to assess the effect of season (hot versus cold) on individual sizes, when the Shapiro and Bartlett’s tests confirmed normality and homogeneity of variance. Otherwise, non-parametric Wilcoxon tests were used.

In order to compare the three sites, the data were either analyzed using one-way analysis of variance (ANOVA) followed by Tukey’s honest significant difference (HSD) post-hoc tests or using Kruskal-Wallis tests depending on whether the data was normally distributed. The chi-square test (χ²) was used to compare the sex ratios and sexual maturity stages.

## 3. Results

### 3.1. Length-weight relationship and condition factor

**Fig 2:**
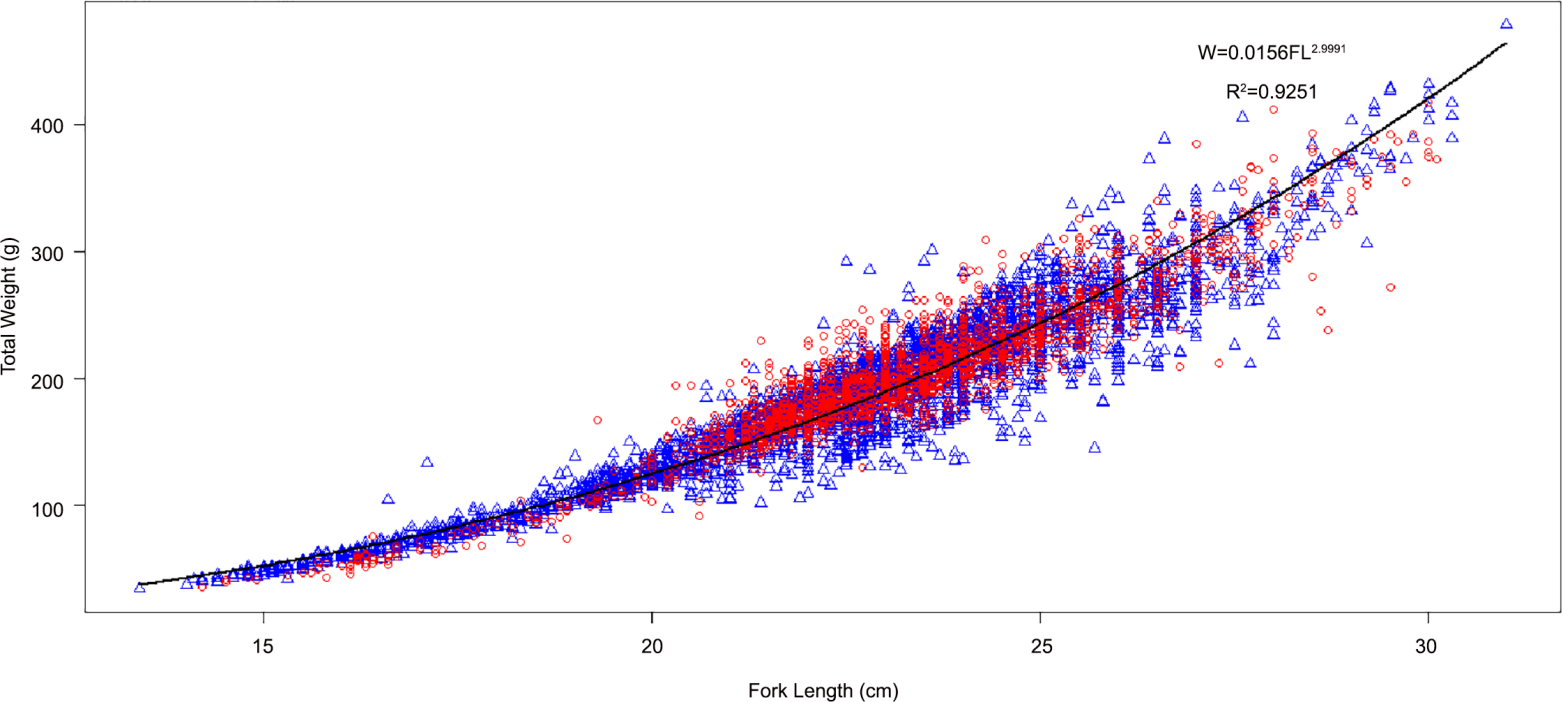
Length-weight relationships for *Sardinella maderensis* in Senegalese waters. Blue triangles and red dots represent the length-weight relationships during the cold season and hot season, respectively. The black line is the model fitted to all measurements regardless of season.

The analysis of the relationship between the total weight and fork length (Eq 1) using a total of 5,660 specimens showed that it was isometric: a = 0.02 ± 0.04, b = 3.00 ± 0.01 and r^2^ = 0.93 (Fig 2). For the hot season only (from June to October), *S*. *maderensis* exhibited negative allometry (a = 0.02 ± 0.07; b = 2.95 ± 0.02; R^2^ = 0.89 and n = 2043) and for the cold season (November to May), growth showed also slightly negative allometry (a = 0.02 ± 0.04; b = 2.98 ± 0.01; R^2^ = 0.94 and n = 3617).

**Fig 3:**
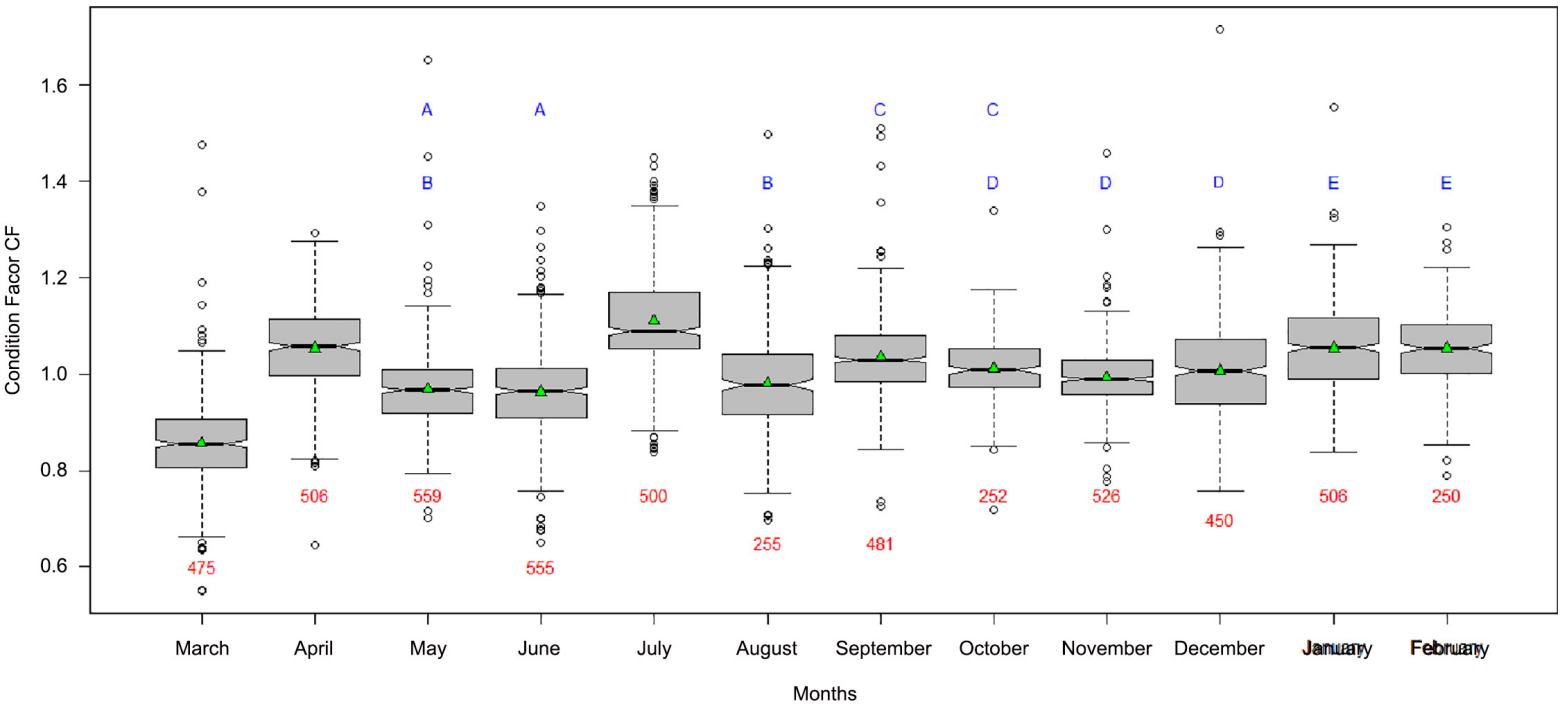
Condition Factor (CF) for *Sardinella maderensis* from March 2012 to February 2013. Means are represented by green triangles inside the notched box plots. Red figures under the error bars are the sample sizes. Months with the same blue letters (A-E) are not significantly different according to the Kruskal-Wallis test.

The monthly variation in the condition factor (CF) is shown in Fig 3. The average CF calculated from a total of 5,660 individuals was equal to 1.01 ± 0.11. Comparisons of CF between months using the Kruskal-Wallis test suggested significant differences (χ² = 1814.64, df = 11, p-value < 0.05), which were confirmed for several monthly pairs by pairwise comparisons using the Kruskal-Wallis Multiple Comparison test (Fig 3). During April and July, the CF was higher CF but no real seasonal trend was identified.

### 3.2. Size spectrum

**Fig 4:**
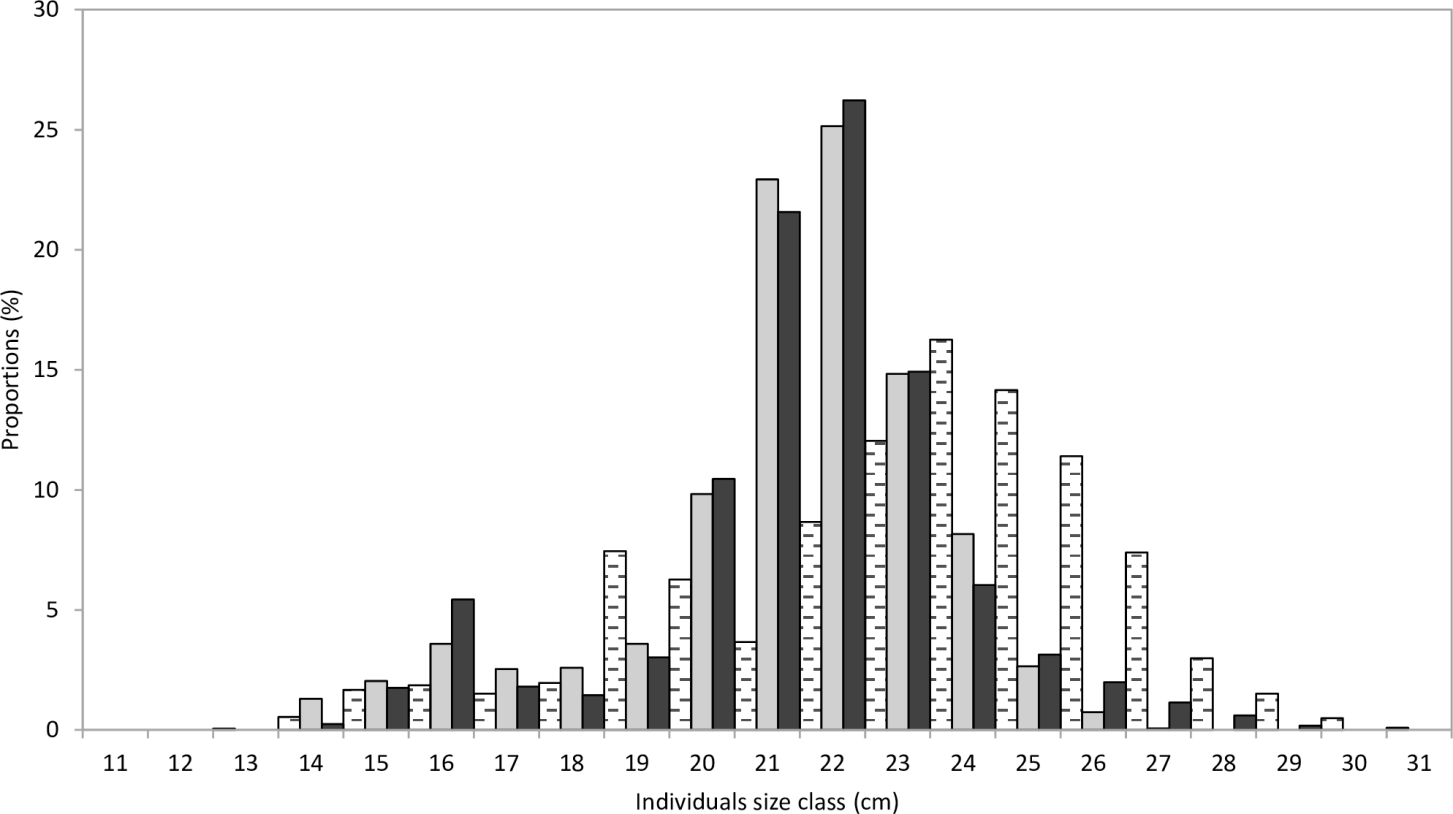
*Sardinella maderensis* size spectra for Kayar (dashed horizontal), Mbour (black), and Joal (grey). Sizes are represented by the fork length.

The length-frequency distributions from Kayar, Mbour and Joal had two modes at each site (Fig 4). The first minor peak length occurred at 19 cm at Kayar and at 16 cm for both Mbour and Joal. The second major peak length occurred at 24 cm at Kayar and at 22 cm for both Mbour and Joal. Individuals caught at “Petite Côte” were smaller than those caught at “Grande Côte” (Fig 1).

### 3.3. Growth parameters

**Fig 5:**
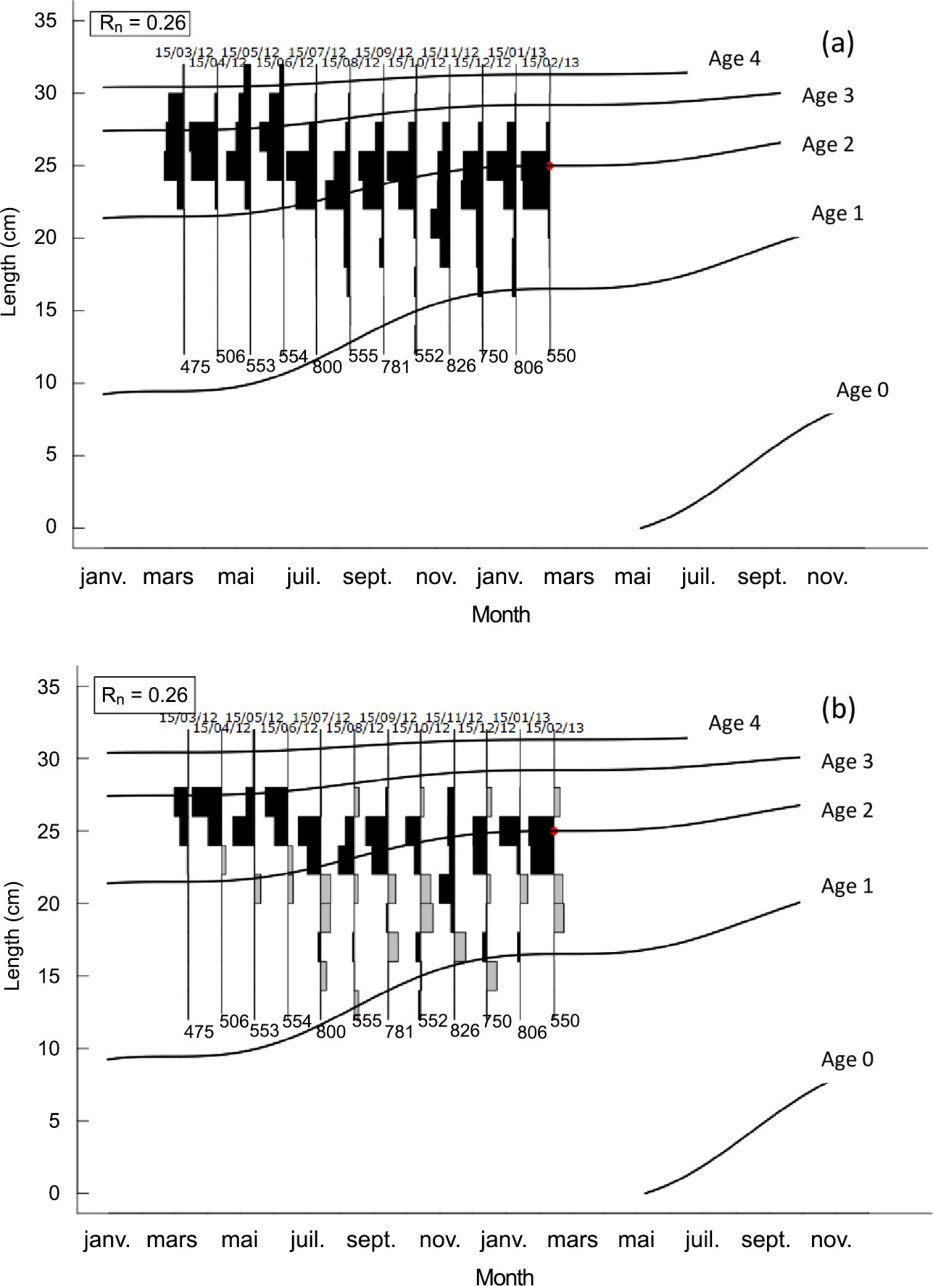
Seasonalized von Bertalanffy growth curve (with L_∞_ = 33.40 cm, K = 0.34 year^-1^, C = 1 year^-1^, WP = 0.15 and R_n_ = 0.26) of *Sardinella maderensis* superimposed over (a) the original length-frequency histograms and (b) the restructured length-frequency histograms. The black and grey bars are positive and negative deviations from the “weighted” moving average of two size classes and represent pseudo-cohorts. The red dot is the starting point through which the curve passes to fit the model by maximizing R_n_ (index of goodness of fit which is analogous, but not equivalent to the correlation coefficient r in linear regression (see Eq 4)).

**Fig 6:**
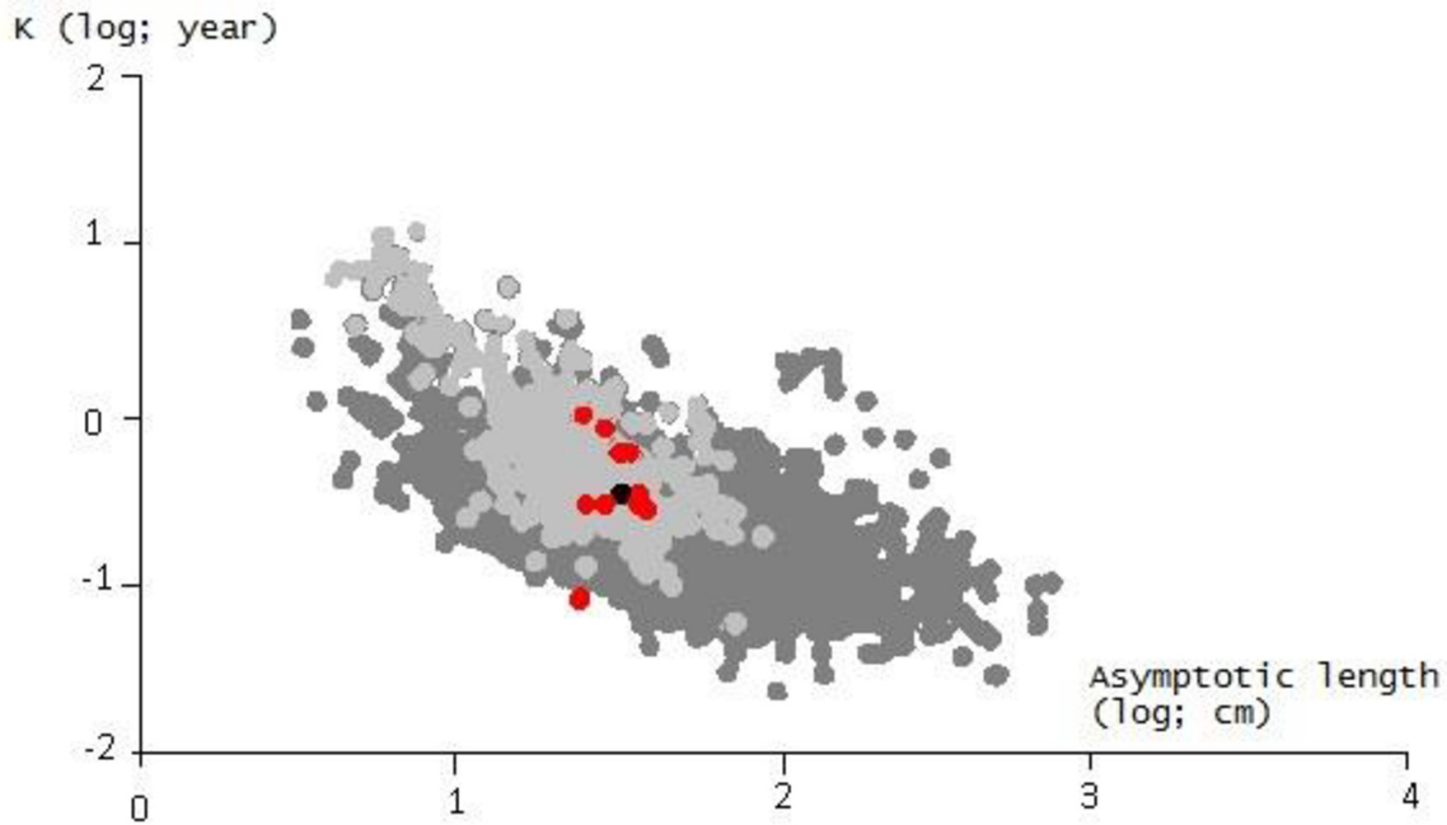
Auximetric plot for *Sardinella maderensis* in Senegalese waters using the data from Table 2. Dots represent the growth performance index (*φ*') fitted against Log_10_ (K) on the y-axis and Log_10_ (L_∞_) on the x-axis. Light grey dots represent fish species in the same family (*Clupeidae*) while dark grey dots are other species. Red dots represent values for *Sardinella maderensis* from the literature and the black dot represents the value from this study.

L_∞_ was estimated at 33.40 cm using the Wetherall method [28] as implemented in ELEFAN in R (S1 Fig). This estimate was used with the fixed values of C and WP giving the best fit value of K = 0.35 year^-1^ (S2 Fig). Fig 5 shows the length-frequency plots in Table 2, which were re-expressed as “peaks” and “troughs”, with the growth curve superimposed. The growth performance index (*φ*'), corresponding to the estimated growth parameters, was 2.59, which is close to the mean *φ*' (Δ = 0.09) for this species (Table 3). The estimated values of L_∞_ and K were plotted on an auximetric plot (Fig 6). The estimated t_0_ value obtained by substituting L_∞_ and K into Eq 6 [23] was -0.46 year. *S*. *maderensis* grows fast in the first year: it measures 13 cm at 6 months and reaches 17 cm after one year (Fig 5). The growth rate decreases gradually with age and reaches 25 cm at 2 years and 29.5 cm at 3 years.

**Table 3:** Growth parameters of *Sardinella maderensis*, based on the results of various publications in West Africa. N/A means not available; L-F means length-frequencies. The length is expressed as fork length.

L_∞_ (cm)	K (year^-1^)		t_0_ (year)	*φ*'	Methods		Country	Source, year
**37.5**	0.30		0	2.63	L-F		Senegal	[30], 1988
**39.5**	0.45		0	2.85	L-F		Senegal	[29], 1988
**30.3**	0.49		-0.59	2.65	calcified structures + L-F		Senegal	[8], 1986
**35**	0.61			2.87	calcified structures		Senegal	[43], 1955
**24.4**	0.08		0.29	1.68	calcified structures		Congo	[42], 1968
**24.9**	0.99		N/A	2.79	calcified structures		Congo	[42], 1968
**39.6**	0.28			2.64	calcified structures		Congo	[44], 1955
**32.5**	0.59		N/A	2.79	L-F		Cameroon	[38], 1989
**27.2**	1.26		-0.85	2.97	L-F		Cameroon	[39], 1995
**27.2**	0.48		-0.06	2.55	L-F		Cameroon	[38], 1989
**29.1**	0.83			2.85	L-F		Cameroon	[40], 1989
**37.5**	0.34		-0.25	2.68	L-F		Nigeria	[41], 1989
**29.6**	0.35		N/A	2.49	L-F		Sierra Leone	[36], 1996
Mean *φ*' of past studies				2.63				
**33.4**	0.35	-0.46		2.59	L-F	Senegal	This study	

### 3.4. Sex ratio and length at first maturity

**Fig 7:**
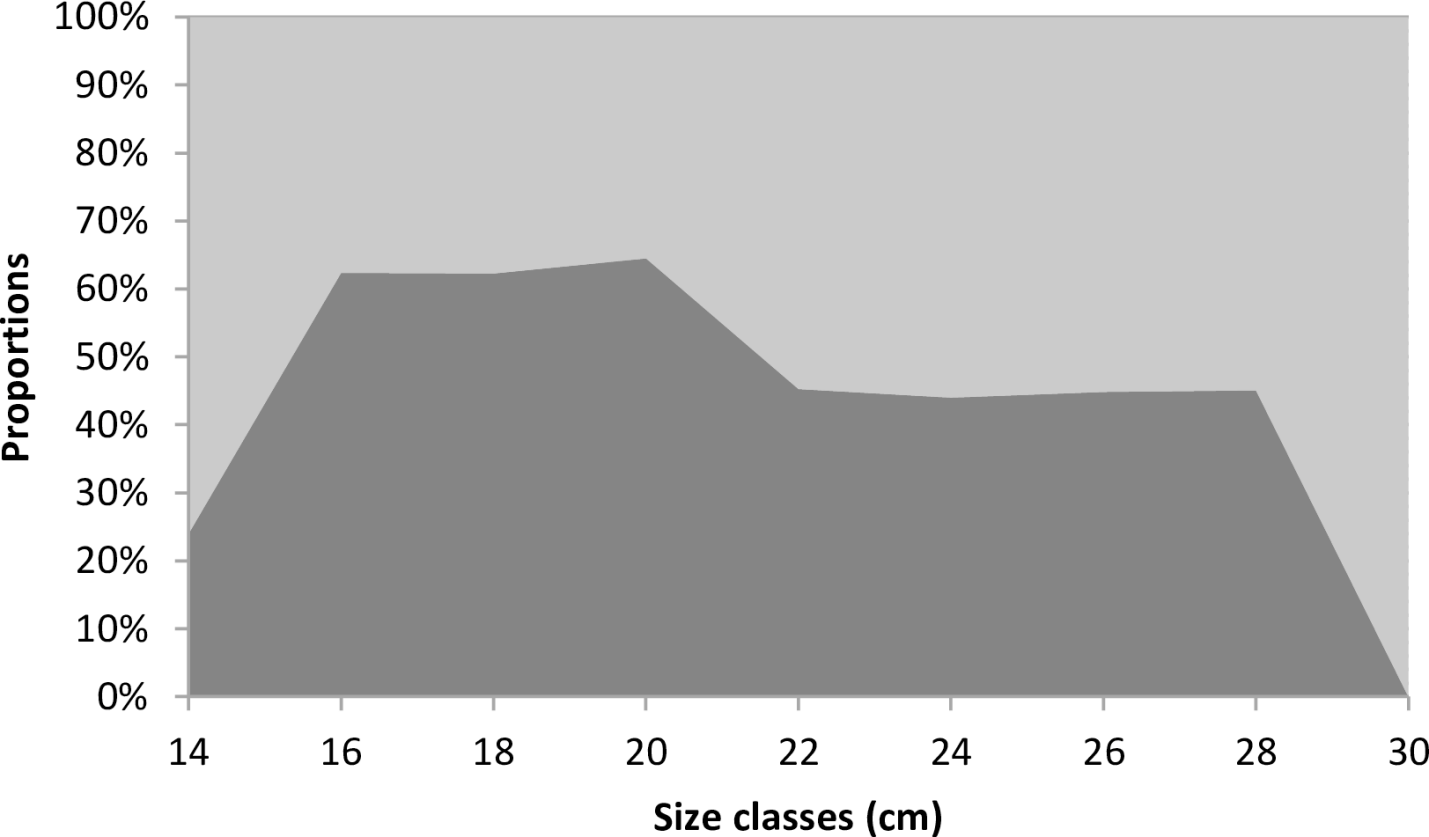
Female (light grey) and male (dark grey) *Sardinella maderensis* sex ratio in 2 cm size classes. Sizes are represented by the fork length.

The sex ratio (SR) for all samples of *S*. *maderensis* was biased in favor of females (55.81%; n = 1068). The proportion of females was significantly higher in the hot season (SR = 58.97%, χ ² = 13.82, df = 1, p-value < 0.05) compared to the cold season (SR = 53.68%, χ ² = 3.45, df = 1, p-value = 0.06). The sex ratio broken down by size class was male-biased between 15 to 22 cm while females dominated under 15 cm and over 22 cm (Fig 7). Broken down by sexual maturity stages, the differences were only statistically significant in stages II and VI (Table 4). In stage VI, all individuals were females (n = 4). In stages I and V, there were more males while there were more females in stages II (p-value < 0.5, n = 166), III and IV.

**Table 4:** The proportions of male and female *Sardinella maderensis* individuals at the different sexual maturity stages.

Stage	Sample size	Males (%)	Females (%)	χ²	Degrees of freedom df	p-value
I	43	53.49	46.51	0.21	1	0.65
II	166	41.57	58.43	4.72	1	< 0.05
III	542	47.79	52.21	1.64	1	0.20
IV	299	45.82	54.18	2.09	1	0.15
V	72	56.94	43.06	1.39	1	0.24
VI	4	0	100	4.00	1	< 0.05

**Fig 8:**
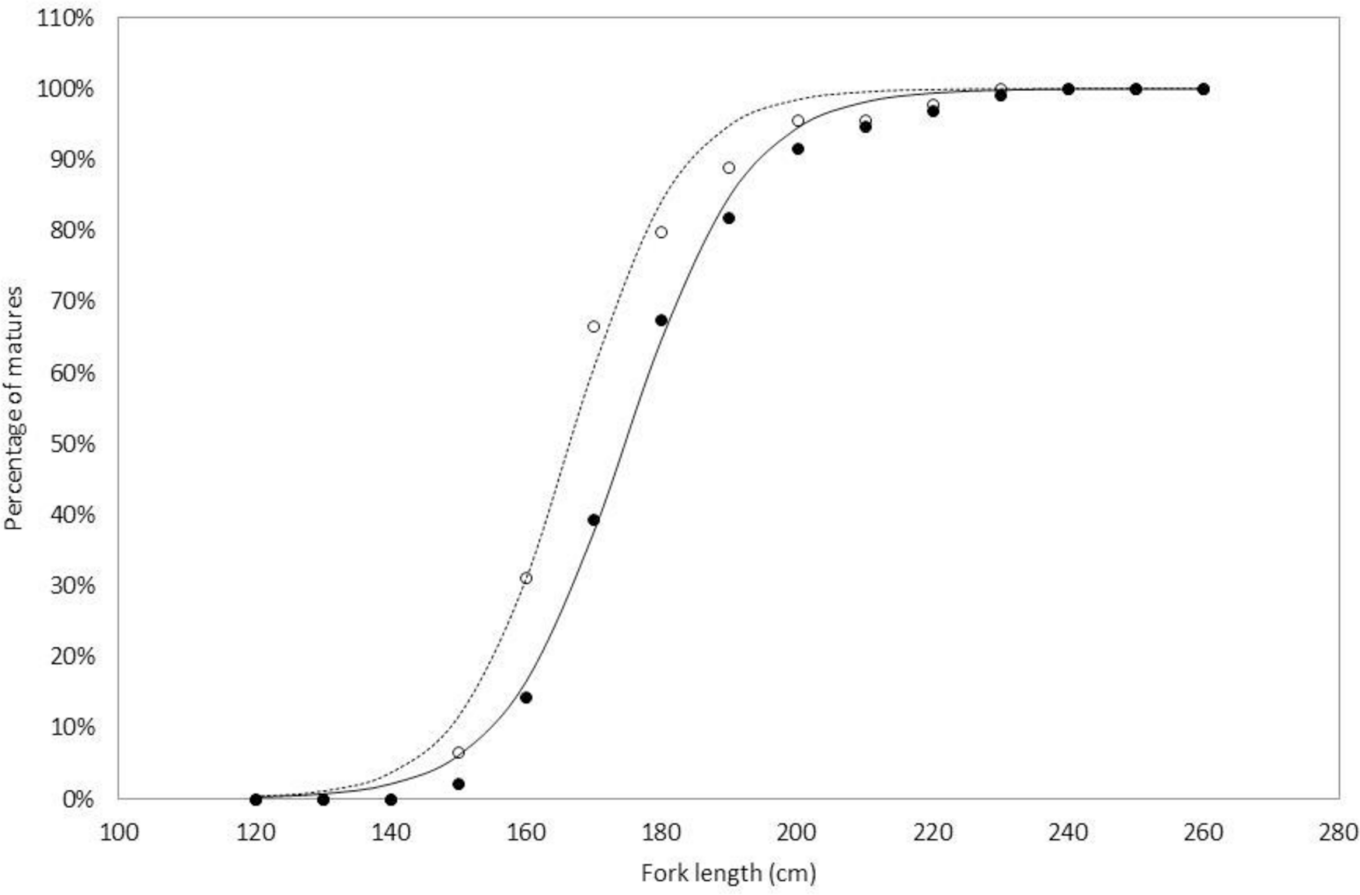
Length at sexual maturity of *Sardinella maderensis*. Measured male (white dots), measured female (black dots). Expected distribution of mature individuals is represented by a solid line for females and a dashed line for males.

The body length of sexually mature females ranged from 12 cm to 26 cm, while that of mature males ranged from 13 cm to 23 cm. The cumulative frequency curve of individuals that had reached or passed maturity stage 3 indicated that nearly 50% (L_50_) of females were mature at 17.50 cm and 50% of males were mature at 16.60 cm (Fig 8).

### 3.5. Spawning seasons

This study confirmed that *S*. *maderensis* spawns throughout the year in Senegal. However, two main reproductive periods can be defined from the analysis of sexual maturity stages and GSI. In this study, the reproductive period was considered to be the whole cycle of gonad maturation up to spawning. The first reproductive period occurred from April to October. Within this period, gonad maturation occurred in April, June and September while spawning occurred mainly from June to August and in October. The analysis of sexual maturity stages showed that individuals in stages I, II, and VI were present in November and December, after the first reproductive period. A second reproductive period, with continuous spawning (more intense than during the first period), occurred from January to the end of February with the gonad maturation period in December.

**Fig 9:**
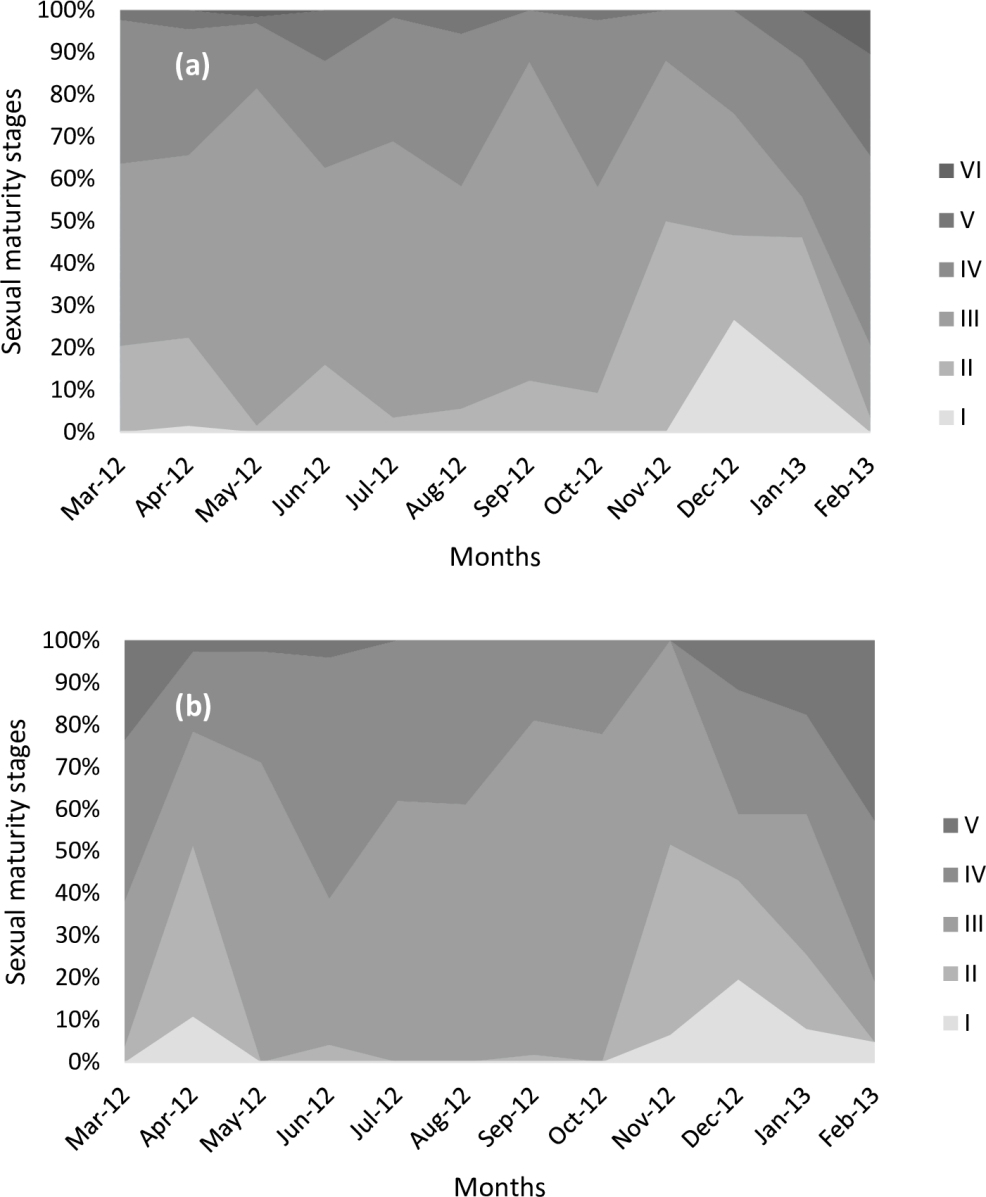
Female (a) and male (b) sexual maturity stages against month of year.

The monthly record of sexual maturity stages showed that females in stage V were more abundant in January-February, June-August, and, to a lesser extent, October. The proportion of females at stages ≥ IV peaked in February, June, August, and October (Fig 9A). Stage III females were mainly present in May and September. Stage V males were present from March to July and from November to February with higher incidence in February. Stage III and IV males were found throughout the year mostly from May to November (Fig 9B).

**Fig 10:**
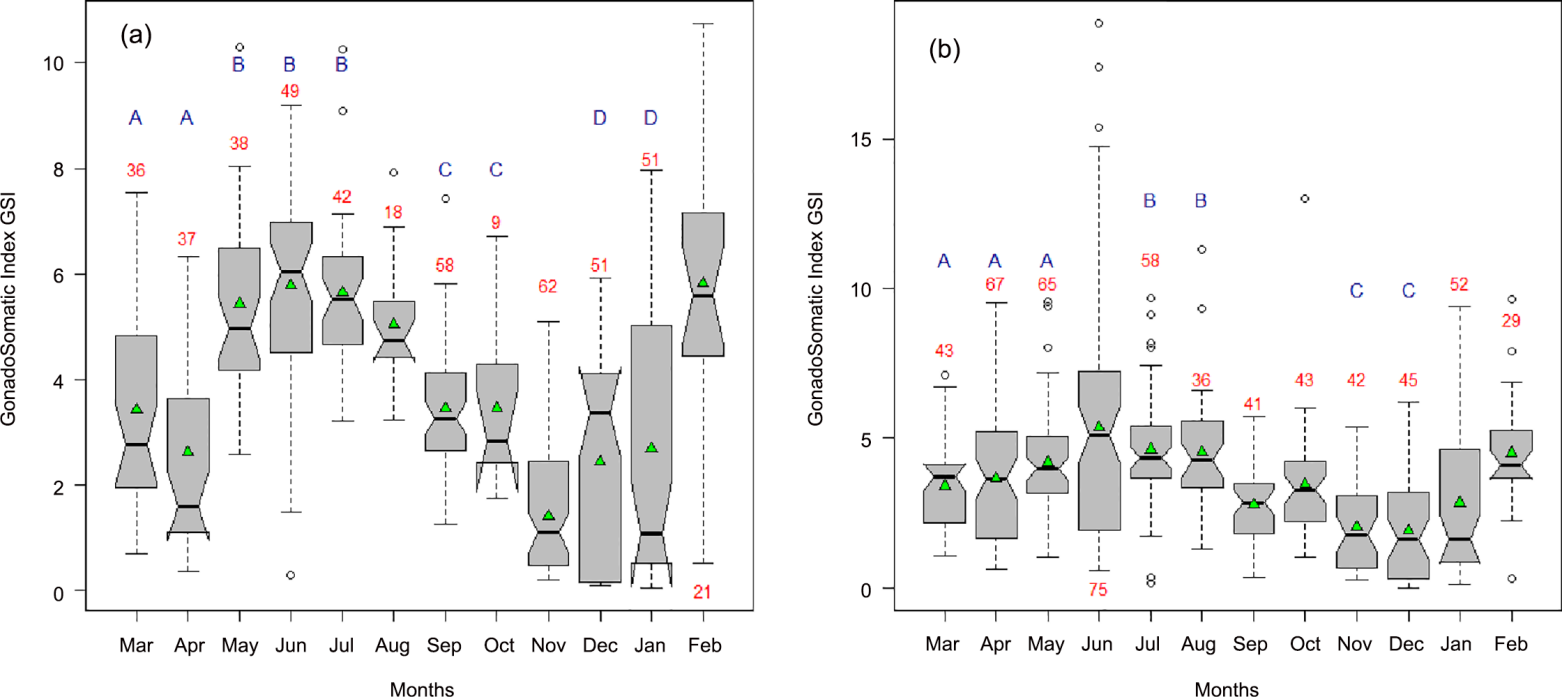
(a) male and (b) female gonadosomatic index (GSI) of *Sardinella maderensis* between March 2012 and February 2013. The means are represented by green triangles inside the notched box plots. Red figures under the error bars are the sample sizes. Months with the same blue letters (A-E) are not significantly different according to the Kruskal-Wallis test.

Monthly changes in GSI (Fig 10) followed sexual maturity stage V (Fig 9). The GSI was significantly different between months both for females (χ² = 115.94, df = 11, p-value < 0.05) and for males (χ² = 203.99, df = 11, p-value < 0.05). GSI was highly variable between August and September and between January and February. There were two spawning peaks in June and February for males and females with lower levels of spawning from June to August. In October, there was a higher presence of stage V and higher GSI simultaneously compared to September and November both for males and females and, therefore, there was probably a short reproductive period. There was a resting period in September and from November to December.

## 4. Discussion

The current study was based on more than 6,000 samples collected along the northern and southern Senegalese coast in 2012–2013 aiming to update the state of knowledge of *S*. *maderensis* and compare findings with previous observations made in Senegal [7–9, 29–31], Mauritania [32, 33], Cabo Verde [34], Gambia [35], Sierra Leone [36], Ghana and Ivory Coast [37], Cameroon [38–40], Congo [41, 42–44] and Nigeria [41].

The other locations where comparable studies have been accomplished had different characteristics: (a) coastal ecosystems without upwelling (Cabo Verde, Sierra Leone, Cameroon and Nigeria), (b) areas where the intensity and duration of the upwelling is different from Senegal (Mauritania, Ghana and Ivory Coast, and Congo) and (c) estuarine ecosystems with either brackish water (the Gambia river [35]) or hyperhaline water (Sine-Saloum estuary, Senegal [31]).

The spatial distribution and exploitation of *S*. *maderensis*, as well as its growth and reproductive patterns, are closely related to the dynamics of the upwelling systems [6]. Adaptation of the biological parameters of the species to environments with different upwelling intensity should be expected. On the northwest African coast, the upwelling is stronger than in the other areas, permanent in the north of Mauritania and from November to May further south in Senegalese waters. The results were also compared with earlier measurements for Senegal to determine whether the biological parameters had changed with an increase in SST of 0.03°C per year over the past 30 years [14] and with the dramatic increase in fishing pressure, with annual landings increasing ten-fold from ~10 000 tons in 1980 to 128 700 tons in 2012 [2].

The growth performance index of *S*. *maderensis* estimated in this study (*φ*' = 2.59) fell in the range (2.40 to 2.97) previously found in Senegal and in the other areas, with the exception of one very low value found in Congo [42] (Table 3). However, it would seem that the low value in the Congo was due to an error because this was the only value outside the range of all other studies including studies in the same area. Our results also confirm other observations in Senegal in 1986 [8] that *S*. *maderensis* reached 17 cm to 18 cm body length at the age of 12 months in Senegal based on scale aging, with the growth rate decreasing until the fish approached their asymptotic length at around 3 years [8]. The results of different methods of aging fish (length-frequency analysis (this study and [8, 29–30]), scales or other calcified structures [8, 42]) converge closely to the same value of the growth performance index. This suggests that this index was not significantly affected by climate variability or increased fishing pressure.

Large individuals (> 26 cm) were common in catches from Kayar (northern coast) but rare in catches from Mbour and Joal (southern coast) (Fig 1). This confirms results obtained more than 25 years ago [8–9]. This is probably due to the migration pattern with larger fish, with greater swimming efficiency, being concentrated along the northern coast where the southward and offshore currents are stronger. Such size segregation in the migration process has been suggested by biophysical modeling in West Africa [45–46] and observed in other places [47–48].

The condition factor is an indication of fish well-being and reproductive activity. This factor was evaluated over the whole year and showed just one trough in March and two peaks, one in April (during the upwelling period) and one in July (period of recovering from spawning). Cury and Fontana [6] suggested that the condition factors are higher in the cold season when upwelling induces plankton blooms. The condition factors were lower during the spawning period and during periods without upwelling.

The allometric scaling exponent reported in this study was similar to that recorded in other studies (Cabo Verde in 1987, Gambia in 2005, Mauritania in 1979 and Senegal in 1982 and 1986; see Table 5). The results also confirmed the measurements obtained in Senegal in 1980 [7] and 1988 [8].

**Table 5:** Length-weight parameters in West Africa for *Sardinella maderensis*. The length is expressed as fork length.

Country	Size range (cm)	a×10^−3^	b	Source, year
Senegal	4–29	10.34	3.14	[9], 1986
Senegal	5–32	9.85	3.17	[8], 1986
The Gambia	9–17	0.07	3.15	[35], 2005
Mauritania	4–29	10.34	3.14	[32], 1979
Senegal	5–27	0.01	3.24	[7], 1980
Cape Verde	7–29	22.93	2.78	[34], 1987
Senegal	13–31	15.60	3.00	This study

The overall sex ratio of *S*. *maderensis* obtained in this study was slightly female biased (55.81%), confirming several previous studies undertaken about 30 years ago in Senegal (Table 6). That was also the case in Cameroun and Mauritania (Table 6), even if locally in the northern coast of Senegal it was reported a sex ratio slightly male biased (~51%). Two different mechanisms have been proposed to explain this. On the one hand, for several fish species, warmer waters increase the female bias [49–50]. This is in line with our observations in Senegal and with an even higher female bias being found in Cameroon (warm waters) but it does not explain the high female bias in Mauritania, where the temperature is usually lower. On the other hand, the “Petite Côte” is a well-known *S*. *maderensis* spawning area [7] and favorable area for fish larval development due to high phytoplankton biomass production and favorable environmental conditions, e.g. high coastal retention for eggs and larvae, in particular during the hot season [51, 52]. Thus in this area and this season, the female bias may be due to an evolutionary strategy giving a better reproduction efficiency for short-lived fish species. This mechanism may also explain the high female bias in Mauritania and Cameroon because the samples were collected in the local reproduction area [6, 7].

**Table 6:** *Sardinella maderensis* sex ratios based on the published literature for West Africa.

Country	Female sex ratio (%)	Source, year
Senegal	55.80	This study
Senegal	56.20	[53], 2010
Senegal	54.60	[8], 1986
Mauritania	59.70	[33], 1988
Cameroon	60.00	[39], 1995

The L_50_ values (16.60 cm for male and 17.50 cm for female) were in the same range as previous studies in West African aquatic ecosystems (Table 7). The L_50_ does not, therefore, appear to be influenced by geographical location [29, 40]. For Senegal, previous estimates of L_50_ were also similar, with the exception of [53] who reported a higher value (expressed in Total Length TL). There did not appear to be any reduction in L_50_ for *S*. *maderensis* for Senegal. However, a reduction in the L_50_ has been observed for *S*. *aurita* off the coast of Ghana [54].

**Table 7:** L_50_ for *Sardinella maderensis* based on the literature for West African areas. L_50_ represents the size at which 50% of individuals in a given population attain maturity. (N/A means not available).

Country	L_50_ male	L_50_ female	Source, year
Senegal	16.60	17.50	This study
Senegal	19.50^a^	20.70^a^	[53], 2010
Ghana-Ivory coasts	N/A	18.50	[37], 1993
Senegal	16.80	17.60	[31], 1996
Senegal	17.00	17.00	[8], 1986
Senegal	17.00	17.00	[7], 1980
Cameroon	17.00	17.00	[45], 1991

a All fish sizes are in cm for fish fork length except (^a^), which is expressed as total length.

This study found two spawning periods for *S*. *maderensis*, confirming previous reports [6]. The first spawning period was from April to October, with two spawning peaks, one in June–August and the other in October. The second spawning period occurred from early January to the end of February, with a single intense spawning peak. Several studies [7–9, 38, 55–56] reported that *S*. *maderensis* almost spawned all year round in Senegal, with two spawning peaks, [one short (~ one month) and intense in cold season (February) and another longer (~four months) in hot season (June to October)], nevertheless we have to notice that it was reported a single spawning period in Mauritania by Pascual-Alayón *et al*. [57] from May to July.

In regard to our results *S*. *maderensis* has only limited plasticity to environmental variations. *S*. *maderensis* is less sensitive to climatic fluctuations and tolerates larger environmental fluctuations than *S*. *aurita*. *S*. *maderensis*, therefore, exhibits a smaller variability of biological and demographic parameters than *S*. *aurita* [6]. *S*. *maderensis* has a less flexible adaptive strategy resulting in a smaller plasticity of the biological parameters studied. This does not allow for better utilization of transitory resources but, on the other hand, the population can persist when conditions are unfavorable.

Previous studies have used the size at first sexual maturity to set the size at first capture for fisheries management [58, 59]. Based on our findings, we recommend that this should be set at 18 cm. The sexual maturity rest period of *S*. *maderensis* should be established in relation to the spawning periods, particularly during spawning from June to October. To draw up a fishery management plan, biological and ecological studies should be expanded to include Mauritania, Gambia and Guinea Bissau. In addition, socio-economic and behavioral aspects of fishing communities must be understood to draw up a management plan that would be acceptable.

The biological parameters of sardinella were expected to change with the effect of climate change and overfishing [13]. It was expected that either the effect of fishing pressure (which has increased considerably in Senegal over the last decades [3]) or the effect of the environment differences would have been detectable. Nevertheless the results of this study are consistent with those previously reported in the literature studied and suggest that *S*. *maderensis* has not been affected by overfishing or global climate change despite its habitat covering very different marine ecosystems including the Canary Current and the Guinea Current Large Marine Ecosystem. This narrow plasticity of the biological parameters studied should be taken into account in fishery management plans.

## Supporting Information

S1 Fig Wetherall [28] plot of *Sardinella maderensis*, leading to an estimate of L_∞_ = 33.40 cm. (DOCX)

S2 Fig K-scan routine for the size-frequency data of *Sardinella maderensis* in ELEFAN (Daniel Pauly, unpublish). (DOCX)

S3 Fig Seasonalized von Bertalanffy growth curve (with L_∞_ = 33.9 cm, K = 0.4 year^-1^, C = 1 year^-1^, WP = 0.15 and R_n_ = 0.21) of *Sardinella maderensis* off the northern coast superimposed over the restructured length-frequency histograms. The black and grey bars are positive and negative deviations from the “weighted” moving average of two size classes and represent pseudo-cohorts. The red dot is the starting point through which the curve passes to fit the model by maximizing R_n_ (index of goodness of fit which is analogous, but not equivalent to r in linear regression (see Eq 4)).
(DOCX)

S4 Fig Seasonalized von Bertalanffy growth curve (with L_∞_ = 32.1 cm, K = 0.38 year^-1^, C = 1 year^-1^, WP = 0.15 and R_n_ = 0.21) of *Sardinella maderensis* off the southern coast superimposed over the restructured length-frequency histograms. The black and grey bars are positive and negative deviations from the “weighted” moving average of two size classes and represent pseudo-cohorts. The red dot is the starting point through which the curve passes to fit the model by maximizing R_n_ (index of goodness of fit which is analogous, but not equivalent to r in linear regression (see Eq 4)).
(DOCX)
