# Supplementary figures and images for: Resilience of Key Biological Parameters of the Senegalese Flat Sardinella to Overfishing and Climate Change
Source: PLoS One. 2016 Jun 9;11(6):e0156143. doi: 10.1371/journal.pone.0156143 (PMC4900567; doi:10.1371/journal.pone.0156143)

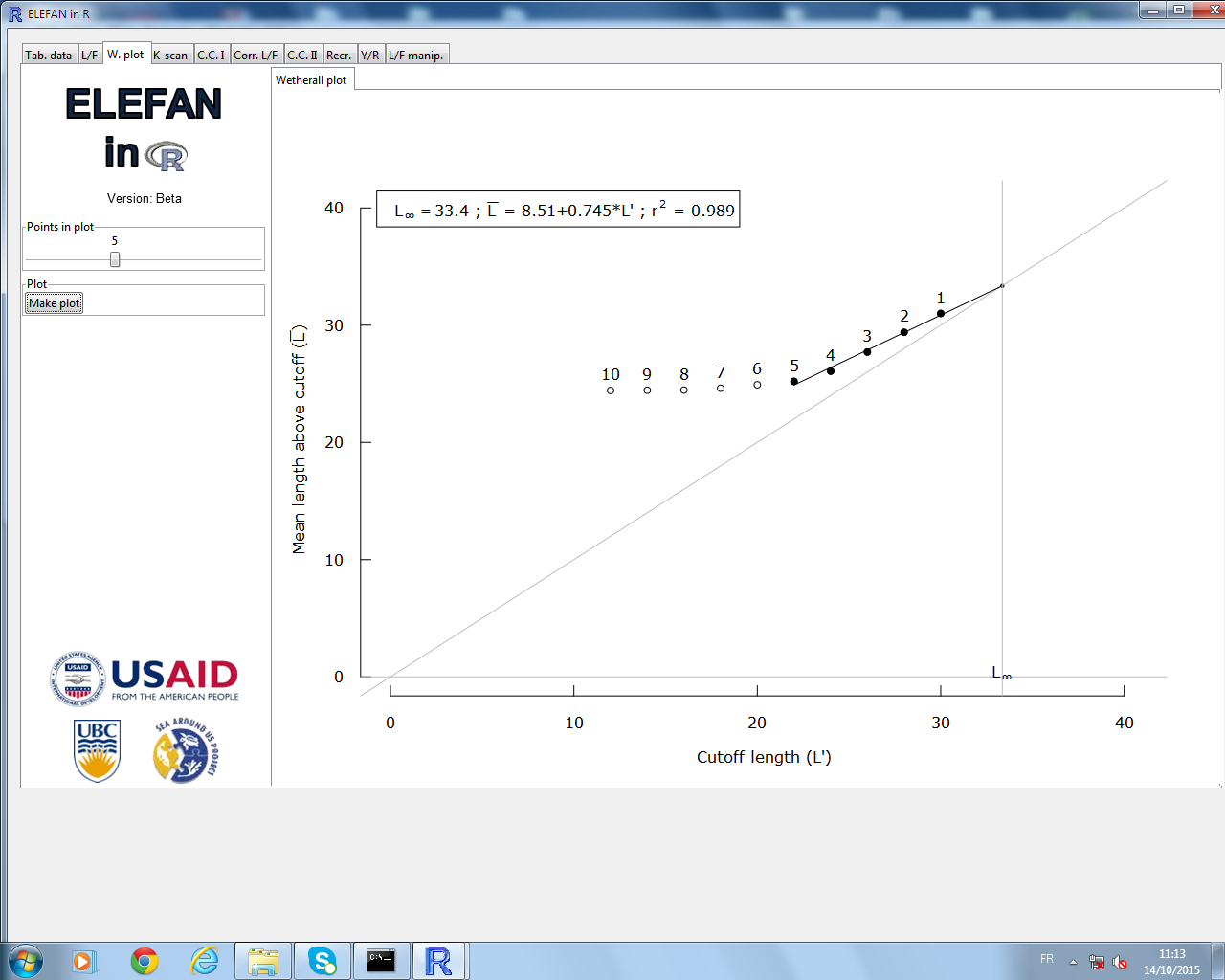


S1 Fig

Supplement: S1 Fig — (DOCX) [file pone.0156143.s001.docx]

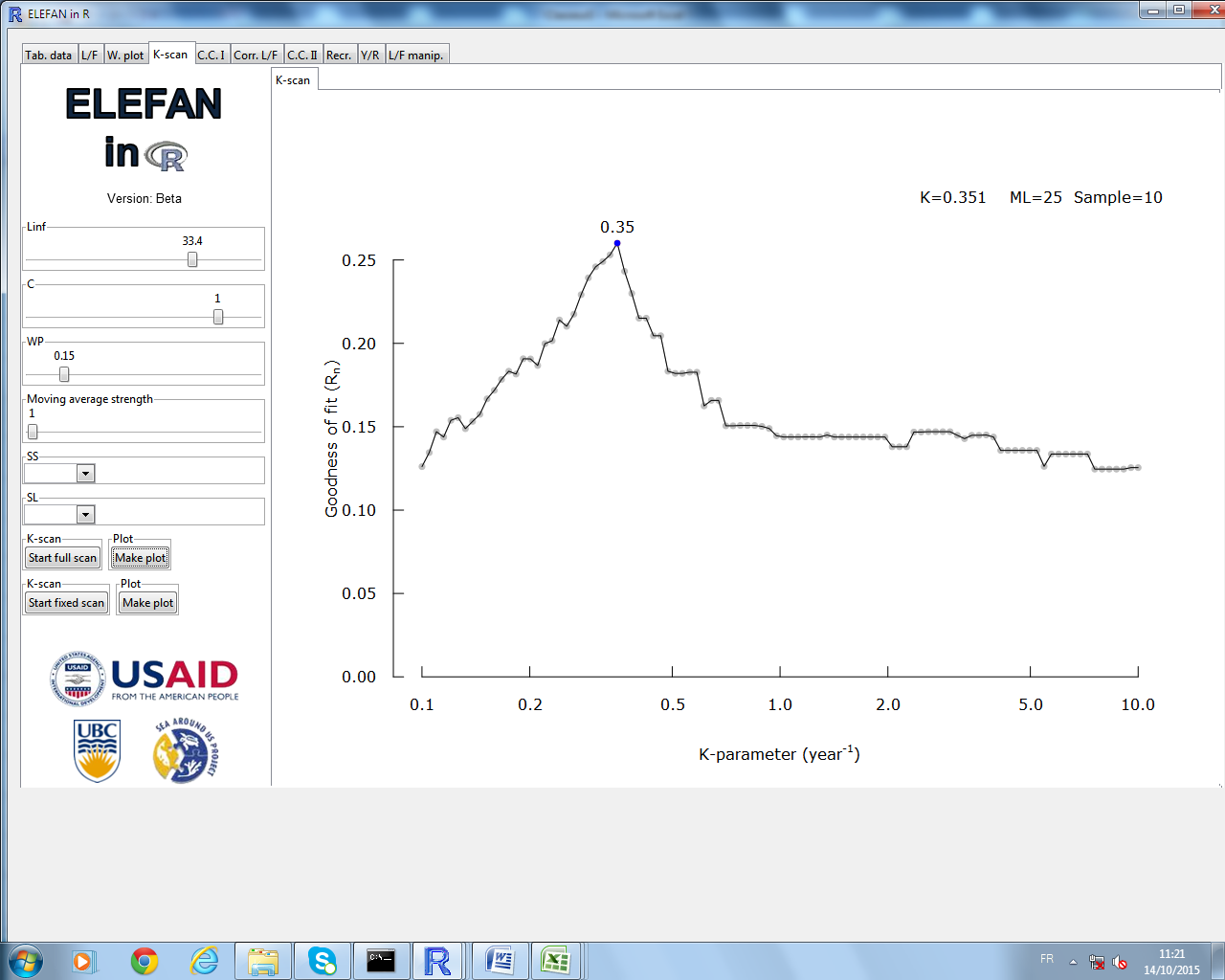


S2 Fig

Supplement: S2 Fig — (DOCX) [file pone.0156143.s002.docx]

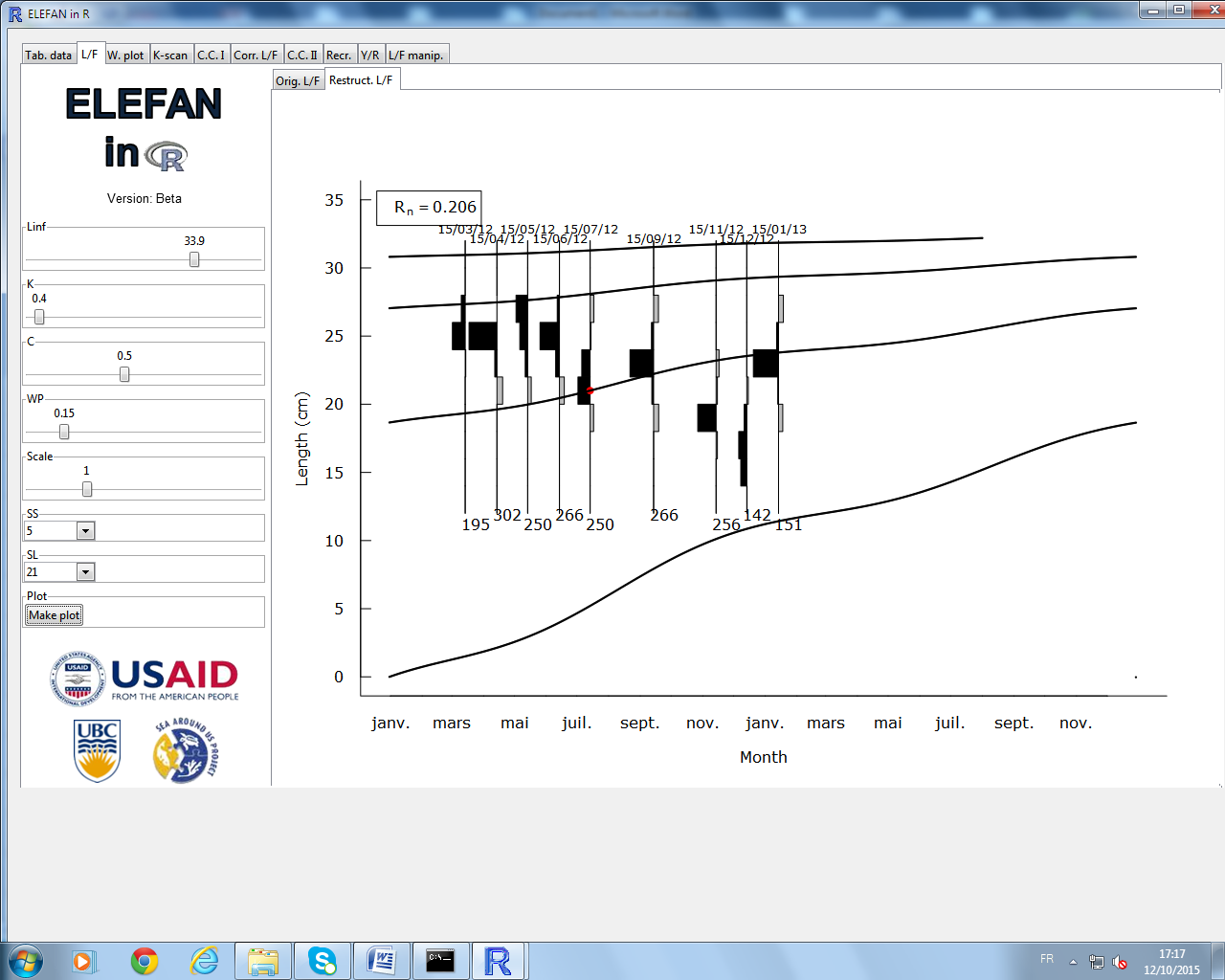


Age 4

Age 3

Age 2

Age 1

S3 Fig

Supplement: S3 Fig — The black and grey bars are positive and negative deviations from the “weighted” moving average of two size classes and represent pseudo-cohorts. The red dot is the starting point through which the curve passes to fit the model by maximizing Rn (index of goodness of fit which is analogous, but not equivalent to r in linear regression (see Eq 4)). (DOCX) [file pone.0156143.s003.docx]

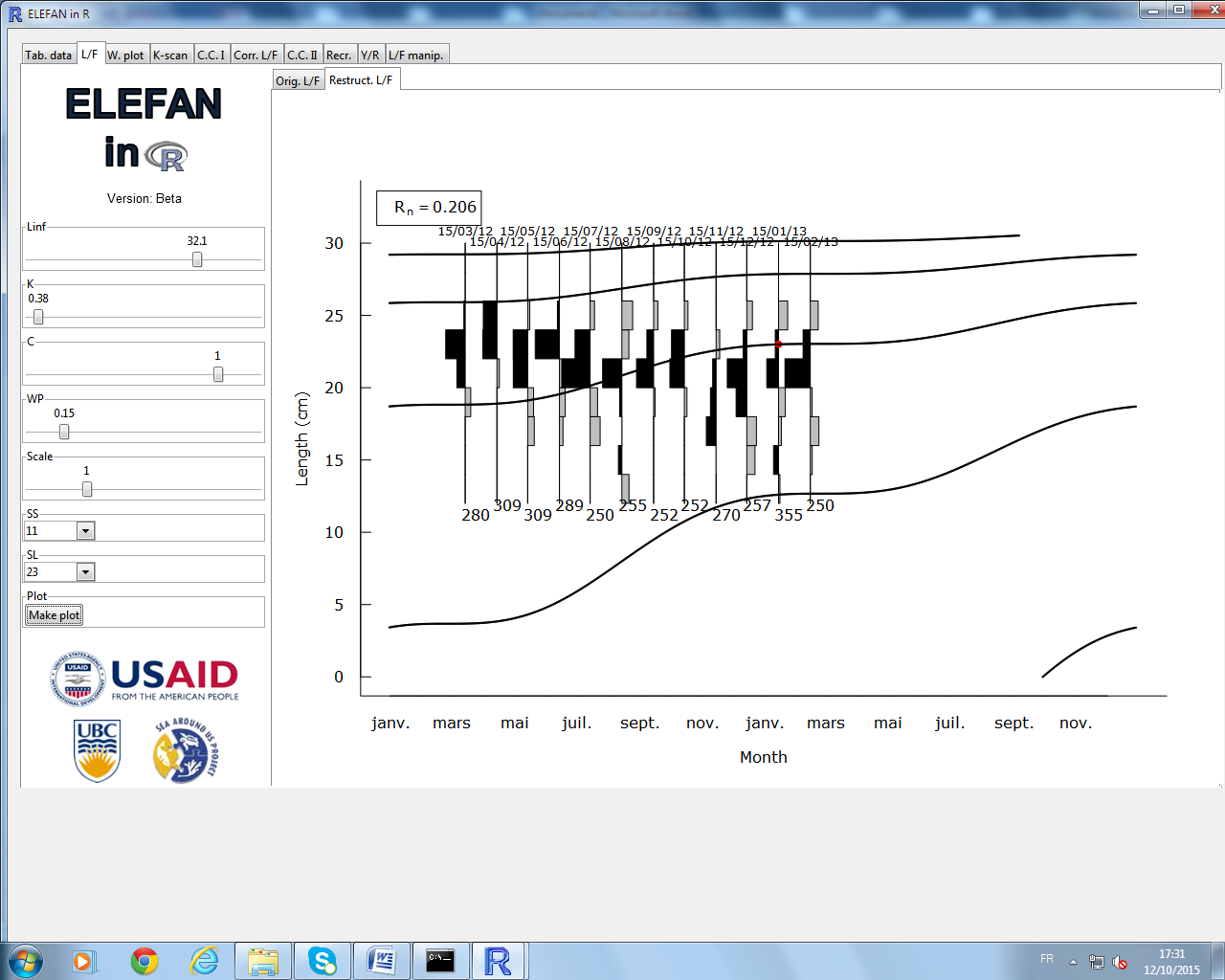


Age 3

Age 4

Age 2

Age 1

Age 0

S4 Fig

Supplement: S4 Fig — The black and grey bars are positive and negative deviations from the “weighted” moving average of two size classes and represent pseudo-cohorts. The red dot is the starting point through which the curve passes to fit the model by maximizing Rn (index of goodness of fit which is analogous, but not equivalent to r in linear regression (see Eq 4)). (DOCX) [file pone.0156143.s004.docx]
